# Transcranial direct current stimulation and cognitive training in the treatment of cognitive deficit in schizophrenia: a randomized controlled trial

**DOI:** 10.1186/s12888-025-07749-5

**Published:** 2026-01-06

**Authors:** Zuzana Stuchlíková, Nina Biačková, Olga Laskov, Anna Čechová, Natálie-Anna Böhmová, Veronika Renková, Barbora Jelínková, Tomáš Novák, Monika Klírová

**Affiliations:** 1 National Institute of Mental Health, Klecany, Czech Republic; 2 3rd Faculty of Medicine, Charles University, Prague, Czech Republic

**Keywords:** Cognition, Cognitive training, Neuromodulation, tDCS, Schizophrenia

## Abstract

**Background:**

Cognitive impairment is a core feature of schizophrenia spectrum disorders and a major contributor to functional impairment in patients with the disorder. Given the limited effects of pharmacological treatments on cognition, there is an increasing interest in the use of alternative therapeutic approaches, including cognitive training and non-invasive brain stimulation. Our study aimed to evaluate the effectiveness of the combined treatment of tDCS and cognitive training on improving cognitive functions in patients with schizophrenia.

**Methods:**

This one-week, randomized, double-blind, placebo-controlled trial explored the effects of the combined intervention of anodal tDCS to the left dorsolateral prefrontal cortex and computer-based cognitive training on cognitive functions in schizophrenia. 30 clinically stable patients, aged 18 to 50 years, with an ICD-10 diagnosis of schizophrenia were randomized into two groups for five daily sessions of cognitive training and sham or active tDCS. Anodal tDCS (2 mA) was applied with the anode over left DLPFC (F3) and the cathode over the right orbitofrontal region (Fp2). Computer-based cognitive training was used. Cognitive testing was performed using the Repeatable Battery for the Assessment of Neuropsychological Status (RBANS, versions A and B).

**Results:**

29 out of the 30 enrolled patients completed the study, and the data was analyzed. Although no statistically significant differences were detected, numerical trends across several RBANS domains (Total Score, Memory, Visuoconstructional, and Language indices) were slightly higher in the active stimulation group. The attention score was numerically higher in the sham group, but also did not reach a statistically significant level.

**Conclusions:**

The combined intervention of tDCS and cognitive training did not demonstrate significant superiority over cognitive training alone. Nevertheless, numerical trends in memory-related domains suggest potential for benefit. These findings contribute to the growing body of research on neuromodulation in schizophrenia, underscoring both the promise and the challenges of implementing combined therapeutic approaches in clinical practice.

**Trial registration:**

The trial was retrospectively registered in the ISRCTN registry (10.1186/ISRCTN13247154) on 18/08/2021.

**Supplementary Information:**

The online version contains supplementary material available at 10.1186/s12888-025-07749-5.

## Background

Cognitive impairment is a core feature of schizophrenia spectrum disorders [1], alongside positive, negative, and affective symptoms. Cognitive deficits often emerge before the first episode of illness (during the prodromal phase), persist through symptomatic remission, and remain relatively stable over time [2]. Generalized impairments across multiple cognitive domains have been reported [3], including deficits in working memory, attention, language, and executive function [4]. Cognitive dysfunction is a major contributor to functional impairment in schizophrenia and a critical barrier to reintegration into daily life [4, 5]. However, currently available antipsychotic medication has only a limited effect on cognitive deficits [6].

On a neurobiological level, altered functional connectivity and abnormal patterns of neuronal activity across multiple brain regions have been consistently reported in individuals with schizophrenia [7–9]. Prefrontal hypoactivity and disrupted intra- and interhemispheric connectivity are closely associated with cognitive deficits observed in the disorder [10, 11]. Neuroimaging studies consistently demonstrate aberrant activation of the dorsolateral prefrontal cortex (DLPFC). A recent review by Li et al. from 2024 [12] reported that both reduced activation and disrupted connectivity of DLPFC correspond with observed executive dysfunction in this population.

Given the lifetime prevalence of schizophrenia, estimated at 6.35 per 1,000 individuals [13], there is a growing need for effective treatment strategies to address cognitive symptoms. Thus, the focus shifted to nonpharmacological intervention methods, including noninvasive brain stimulation techniques and cognitive training.

Cognitive training is an intervention proposed to reduce the cognitive and functional impairments observed across a range of psychiatric disorders. It involves a systematic repetition of structured tasks designed to target one or more specific cognitive functions with the aim of generalizing benefits and transferring the improvements into non-trained cognitive domains as well [14]. Cognitive exercise, or cognitive training, can be computer-based or “traditional”, using non-computer-based training tasks and activities. In combination with therapist-guided facilitation, strategy development, and mechanisms that promote the generalization of skills to everyday functioning, it represents one of the four core elements of the evidence-based intervention known as cognitive remediation, as defined by Bowie et al. [15]. Meta-analyses suggest that the functional benefits of cognitive remediation are significantly enhanced when delivered in conjunction with broader psychosocial rehabilitation programs [16, 17]. Furthermore, both computerized and traditional forms of cognitive rehabilitation and training have been shown to yield measurable cognitive improvements in individuals with schizophrenia [18].

Neural correlates of cognitive remediation and training include increased activation in the DLPFC [19], inferior frontal gyrus [20], and parietal areas [21], which correspond to improvements in executive function and working memory. Cognitive remediation has been associated with altered functional and structural connectivity [22–25]. Neuroprotective effects and even an increase in gray matter volume following cognitive remediation have also been described [26, 27]. Although increased prefrontal activation was the most common finding in the meta-analysis by Mothersill and Donohoe [28], the effect of cognitive training on specific brain regions likely reflects the type of training employed.

As another nonpharmacological approach, noninvasive brain stimulation has been established as an effective therapeutic modality for various neuropsychiatric conditions [29–31]. Transcranial direct current stimulation (tDCS) is a noninvasive method that uses low-intensity direct current applied through scalp electrodes to modulate neuronal activity and promote neuroplasticity [32–34]. In addition to the immediate effects of stimulation, tDCS can induce lasting post-modulation changes through BDNF-dependent synaptic plasticity, alterations in neuronal connectivity, and regulation of neurotransmitter systems, particularly glutamatergic and GABAergic pathways [32–34]. The clinical effect of neuromodulation is determined by the electric field distribution corresponding to the electrode placement. TDCS thus provides a promising neuromodulation technique capable of targeting hypoactive prefrontal regions and modulating associated neuronal networks [35].

Given its central role in higher-order cognition, the DLPFC represents a potential target for neuromodulation strategies aimed at ameliorating cognitive deficits in schizophrenia. Accordingly, several studies have reported promising results for cognitive enhancement after tDCS with anodal placement over the left DLPFC [36–39]. Supporting this approach, a recent large-scale randomized controlled trial demonstrated significantly greater improvement following tDCS over the DLPFC compared to sham stimulation [40]. However, the research findings have been inconsistent as some randomized trials reported null findings [41–44]. Although an earlier meta-analysis showed positive results and demonstrated a statistically significant improvements in working memory and attention in patients with schizophrenia after tDCS [31], a subsequent meta-analysis focusing on neurostimulation methods for cognitive improvement specifically associated with schizophrenia [45] and a meta-analysis narrowing its scope to tDCS application in this indication [46] did not yield statistically significant results to support the benefits of neurostimulation.

Importantly, the effects of neuromodulation strategies like tDCS may be enhanced when paired with task-specific activation of the targeted brain region. Evidence from several studies indicates that concurrent cognitive or physical training amplifies the therapeutic benefits of tDCS relative to stimulation alone [47–49]. A combination of cognitive training and tDCS also offers a potential synergistic effect on neuronal networks. A recent meta-analysis by Burton et al. from 2023 [50], including research in various neuropsychiatric disorders, reported a small but statistically significant effect of combined cognitive training and tDCS on attention and working memory.

In this context, the present study investigated the efficacy of a combined intervention involving tDCS and computer-based cognitive training in addressing schizophrenia-related cognitive impairments. To date, relatively few studies have implemented cognitive training during each tDCS session in patients with schizophrenia, and the findings have been heterogeneous and inconclusive [43, 51–55]. We hypothesized that the combined intervention would result in a significantly higher improvement in cognitive performance compared to cognitive training alone. Further, our study only included subjects with the ICD-10 diagnosis of schizophrenia, excluding schizoaffective disorder and acute psychotic disorder to narrow the specificity of the sample.

## Methods

### Study overview

This double-blind, parallel group randomized controlled trial was conducted by the National Institute of Mental Health (NIMH) in the Czech Republic between June 2018 and March 2025. Participant enrollment occurred from November 2019 to January 2025. The study sites included the NIMH and the Hospital Ceske Budejovice a.s. Recruitment was conducted through referrals from outpatient clinicians, as well as from inpatient and outpatient services at the study centers and the Daily Sanatorium Ondrejov. The trial was registered in the ISRCTN registry (10.1186/ISRCTN13247154) and received ethical approval from the Ethics Committees of both the NIMH (ref.n.16/21) and the Hospital Ceske Budejovice (ref.n.104/21). Written informed consent was obtained from all participants. The study adhered to the principles of the Declaration of Helsinki. The study adhered to the CONSORT (Consolidated Standards of Reporting Trials) guidelines.

### Participants

Thirty participants were recruited from the study centers based on referrals from attending psychiatrists or psychologists. Inclusion criteria were as follows: (1) Male or female inpatients or outpatients aged 18–50 years; (2) Diagnosis of schizophrenia (ICD-10: F20); (3) Capacity to understand and provide informed consent; (4) Stable and adequate antipsychotic treatment (monotherapy or combination) for at least two weeks prior to enrollment, with no planned changes during the study if clinically appropriate; (5) Mild to moderate symptom severity, defined as a Positive and Negative Syndrome Scale (PANSS) score ≤ 75 at baseline [56]. Inpatients were eligible only if clinically stable at enrollment with no medication changes within the past 2 weeks and no acute behavioral instability.

Exclusion criteria included: (1) Axis I or II psychiatric comorbidity (DSM-IV) within six months prior to enrollment; (2) Contraindications for transcranial direct current stimulation (tDCS), including skin disease, skull fracture, epilepsy, or presence of metallic implants in the stimulation area; (3) Inadequate psychosis treatment according to clinical guidelines; (4) Pregnancy or breastfeeding; (5) Severe or unstable somatic disorders (e.g., cardiovascular disease, neoplasms, endocrine disorders); (6) History of electroconvulsive therapy within three months before enrollment or presence of a neurological disorder (e.g., epilepsy, head trauma with loss of consciousness); (7) High suicide risk, as assessed by the treating psychiatrist; (8) Substance use disorder within the past year, except for nicotine dependence; (9) Sensory or motor impairments preventing participation in cognitive training.

Inadequate psychosis treatment was defined as a failure to meet guideline-recommended antipsychotic dosage or treatment duration according to the Czech guidelines of the Psychiatric Society of the Czech Medical Association of J. E. Purkyně [57].

Participants were randomly assigned (1:1) to either the active or sham tDCS group. Both groups received identical cognitive training. Randomization was performed by M.K. using an unrestricted permuted block design. Participants remained blind to group allocation throughout the study. As per the inclusion criteria, all participants were on a stable medication regimen for at least two weeks before enrollment, and no dose or medication changes were permitted during the trial.

### Intervention

Participants were randomly assigned to two intervention groups, with fifteen participants in each group. All the participants received active cognitive training combined with either active anodal tDCS in the first group or sham tDCS in the second group. The intervention was administered once daily for five consecutive weekdays (1 week), with each session lasting 60 min. Cognitive training was conducted throughout the whole session, while tDCS (active or sham) was applied for the first 30 min. At the end of the first week, patients were given the option to continue the stimulation and cognitive training for an additional two weeks, following the same study design while maintaining blinding.

#### *Cognitive training*

Cognitive training was delivered using the computer-based RehaCom program (HASOMED GmbH), a standardized software platform designed for cognitive rehabilitation. RehaCom includes more than 20 game-like training modules targeting specific cognitive functions, as well as more complex tasks addressing multiple domains. For this study, four modules were selected, each administered for 15 min. Three modules targeted attention (“Attention and Concentration,” “Reaction Behavior,” and “Divided Attention”), and one module targeted working memory (“Working Memory”). All participants completed the same four modules in a set order during each session, resulting in 60 min of training per session. Task difficulty was automatically adjusted by the software according to individual performance. Training was conducted on a laptop or desktop computer. A supervising researcher provided instructions before the first session and was available for assistance during all the following sessions as well. The daily, brief-session format adhered to the manufacturer’s recommended use. Additional information about the RehaCom system is available at https://hasomed.de/en/.

#### *tDCS*

tDCS was administered using the HDCStim programmable stimulator (Newronika, Italy). Electrodes were positioned bilaterally according to the EEG 10–20 system, with the anode over the left dorsolateral prefrontal cortex (F3) and the cathode over the right orbitofrontal region (Fp2). Stimulation was delivered through squared rubber electrodes (25 cm²). Conductive EEG gel was applied to the surface of the electrodes before they were covered with saline-soaked sponges. The electrodes were held in position by an elastic cap. The active tDCS condition involved a 2-mA current applied for 30 min, with initial ramp-up and final ramp-down of current intensity lasting 30 s each. The sham stimulation used an identical electrode assembly and involved only an initial 30-second ramp-up phase immediately followed by a 30-second ramp-down phase to simulate sensations similar to active stimulation. Then the stimulation was stopped and was followed by a 29-minute rest. Sensations were assessed after stimulation to confirm blinding effectiveness and to note any side effects. Investigators administering tDCS were blinded to the group allocation throughout the whole protocol.

**Fig. 1:**
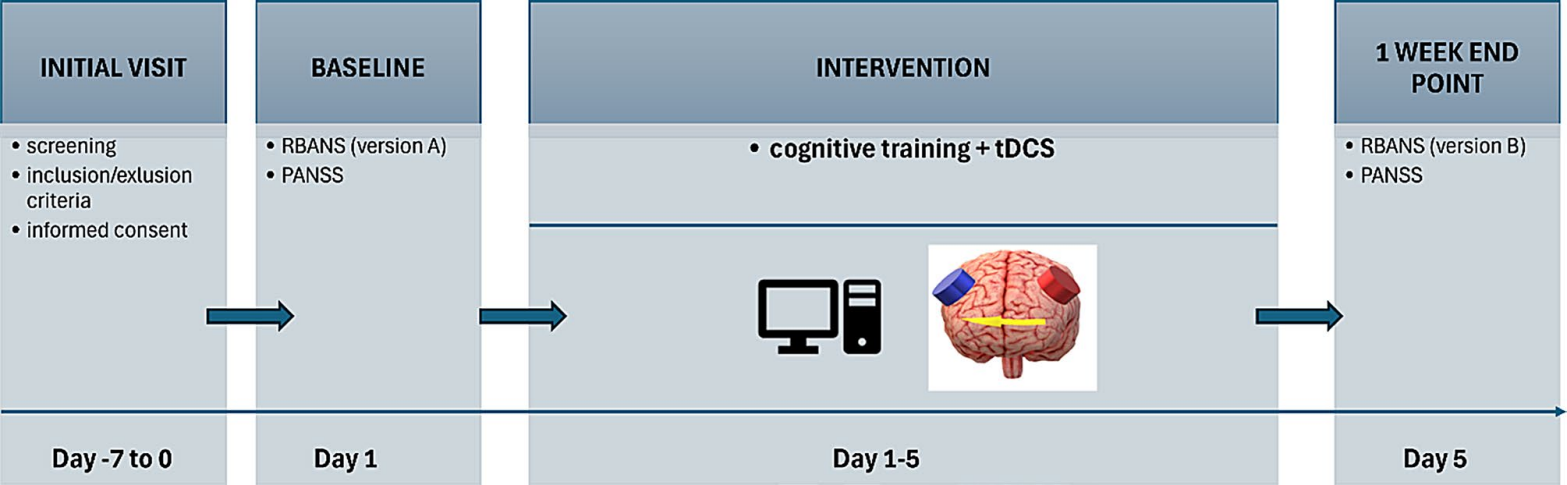
Study design. PANSS – Positive and Negative Syndrome Scale; RBANS – Repeatable Battery for the Assessment of Neurological Status; tDCS – transcranial Direct Current Stimulation

The visualization of the study design can be found in Fig. 1.

### Outcomes and assessment

#### *Primary outcomes*

The primary outcome was the change in the Repeatable Battery for the Assessment of Neuropsychological Status (RBANS) [58, 59] score, used to evaluate cognitive function. Total scores and separate indices, including Immediate Memory, Visuospatial Skills, Attention, Language, and Delayed Memory scores, were analyzed.

#### *Secondary outcomes*

The secondary outcome was the change in symptom severity, assessed using the Positive and Negative Syndrome Scale (PANSS) [60]. Total scores and PANSS positive, negative, and general psychopathology subscale scores were analyzed.

#### *Assessment*

All assessments were conducted by trained investigators using standardized evaluation protocols to ensure consistency and reliability. The evaluating investigator remained blinded to group allocation throughout the study. During the baseline visit, eligibility was determined through clinical evaluation of inclusion and exclusion criteria, and the diagnosis of schizophrenia (ICD-10; F20) was confirmed. Comprehensive clinical and demographic information was obtained, including psychiatric history, current medication, and illness duration. Cognitive performance was assessed using the RBANS, with Version A administered at baseline and Version B at the one-week endpoint. Symptom severity was evaluated using the PANSS at both time points. For participants who elected to continue in the optional extension phase, RBANS Version C and PANSS were administered again at the three-week endpoint. The three-week assessments were optional and were analyzed as exploratory outcomes.

### Statistical methods

To assess the differences between the active and sham stimulation groups, both parametric and non-parametric statistical tests were used depending on the distribution and nature of the data.

To compare the effects of active and sham stimulation on cognitive performance, we used analysis of covariance (ANCOVA) with group (active vs. sham) as a between-subject factor and the baseline score (V0) as a covariate. This allowed us to control for small baseline differences and to test whether the groups differed at the 1-week endpoint (V1) after adjustment. ANCOVA was performed for all RBANS indices, including Immediate Memory, Visuospatial/Constructional, Language, Attention, Delayed Memory, and Total Score. For each domain, we report the F statistic, p-value, and partial η² (effect size). Adjusted means (95% CI) were computed for both groups.In cases where the assumption of normality was not met or ordinal data were analyzed, the non-parametric Wilcoxon rank-sum test was applied. This approach was used, for instance, for PANSS scores where the distribution was non-normal or not suitable for parametric testing.

All statistical analyses were conducted in R (version 4.3.0), and a significance threshold of *p* < 0.05 was applied.

## Results

### Participants

**Fig. 2:**
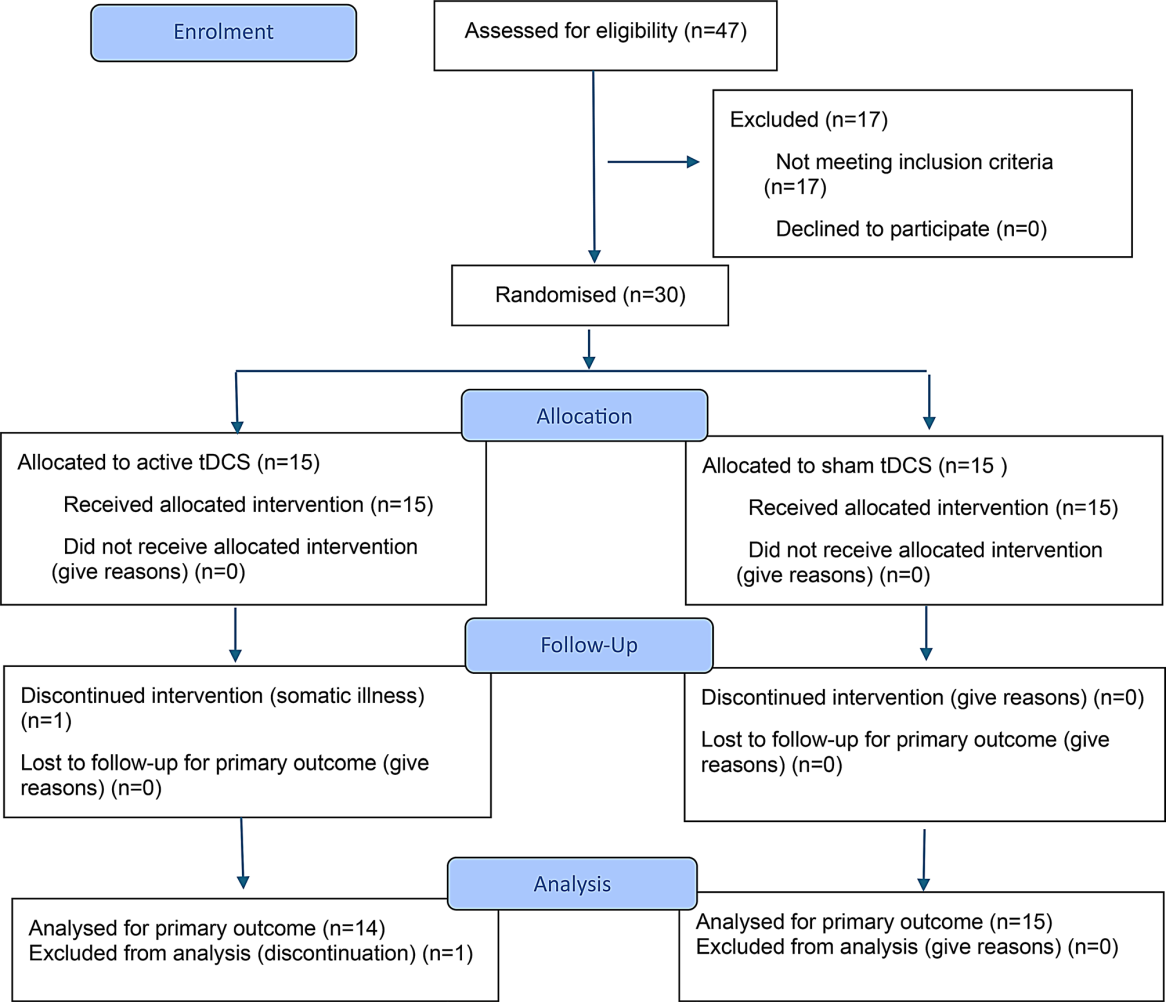
CONSORT 2025 Flow Diagram. Flow diagram of the progress through the phases of the randomised trial (that is, enrolment, intervention allocation, follow-up, and data analysis)

A total of 47 patients were assessed for eligibility. 17 patients were excluded due to not meeting the inclusion criteria, primarily because of medication changes within the two weeks preceding study enrollment. 30 patients were ultimately included in the trial and randomized to the two study groups. 29 out of 30 participants were included in the statistical analysis. One participant in the active group dropped out during the intervention period due to an unrelated acute respiratory infection. A flow diagram of the randomized trial’s progress can be found in Fig. 2. The two groups did not statistically differ in demographic data; specifically, gender (χ² = 0.60, *p* = 0.439), age (t(27.689) = 0.41827, p-value = 0.679), and illness duration (t(24.82) = 0.790, p-value = 0.437) were evaluated (Table 1).

**Table 1:** Baseline demographic data

	Active group (*n* = 15)	Sham group (*n* = 15)	*p*-value
Age	34.933 (9.640)	33.533 (8.667)	0.679
Gender (Female: Male)	4:11	6:9	0.439
Illness Duration	9.214 (7.402)	11.286 (8.668)	0.437
OLZ-eq mg/day	20.72 (8.74)	23.76 (12.02)	0.435
Education			
Elementary Vocational High School University	3 3 6 3	1 0 12 2	0.102
Somatic Illness			
Hypertension Arrythmia Hyperlipidemia Thyroid Autoimmune Neurological Sleep apnea	1 0 2 1 0 1 0	1 1 3 2 1 1 1	-
Employment			
Employed Unemployed Disability	3 4 8	2 2 11	0.119

### RBANS

#### *Baseline visit*

The difference between the active and sham groups at the baseline was statistically significant in the RBANS Total Score (p-value < 0.001), the Visuospatial Score (p-value < 0.001), the Delayed Memory Score (p-value = 0.001), and the Attention Score (p-value < 0.001). See Table 2.

**Table 2:** RBANS and PANSS change during the study

tDCS		Active (*n* = 14)		Sham (*n* = 15)		P-values	
		Baseline (V0)	One-week endpoint (V1) mean	Baseline (V0)	One-week endpoint (V1) mean	Active vs. Sham (V0) difference	Active vs. Sham (V1-V0) Difference
PANSS	Total	42.07 ± 18.55	40.71 ± 18.50	42.07 ± 19.51	38.20 ± 17.51	0.548	0.19
	Negative	15.83 ± 4.17	15.00 ± 3.57	12.46 ± 2.6	11.62 ± 2.10	0.01	0.989
	Positive	9.75 ± 2.7	9.50 ± 2.65	10.54 ± 3.73	10.08 ± 3.23	0.68	0.644
	General	23.5 ± 3.09	23.00 ± 3.16	25.54 ± 4.99	22.38 ± 5.58	0.23	0.031
RBANS	Total	82.36 ± 18.85	88.14 ± 11.72	85.8 ± 14.9	89.13 ± 8.97	< 0.001	0.253
	Immediate Memory	82.71 ± 18.99	92 ± 18.7	84.87 ± 13.86	88.3 ± 15.3	0.92	0.222
	Visuospatial Skills	95.71 ± 18.0	98.14 ± 16.29	92.6 ± 15.9	100.53 ± 13.13	< 0.001	0.967
	Speech	93.43 ± 11.77	95.29 ± 7.78	91.27 ± 10.53	92.7 ± 7.16	0.76	0.355
	Attention	75.21 ± 18.07	78.93 ± 14.74	80.8 ± 12.71	86.87 ± 12.03	< 0.001	0.174
	Delayed Memory	85.57 ± 19.35	92.36 ± 15.87	89.47 ± 17.97	92.87 ± 15.47	0.001	0.682

Table 2 provides data for active and sham groups in RBANS and PANSS scores at Baseline visit (V0) ± standard deviation; mean ± standard deviation at one-week endpoint (V1); p-values in difference between active and sham group at point V0 and at V1-V0

PANSS – Positive and Negative Syndrome Scale; RBANS – Repeatable Battery for the Assessment of Neurological Status; tDCS – transcranial Direct Current Stimulation

#### *One week end-point visit*

When baseline scores were included as covariates, no significant group differences were observed in any RBANS domain or the total score (all *p* > 0.10). Although the active tDCS group showed slightly higher adjusted means across several cognitive domains, these effects were small (partial η² < 0.07) and did not reach statistical significance. Both groups showed numerical improvement after one week of combined training, suggesting a general practice effect rather than a stimulation-specific one. Detailed ANCOVA results and adjusted means (95% CI) are shown in Table 3.

**Table 3:** RBANS ANCOVA results

Scale	F	*p*	Partial η²	Adjusted mean sham	Adjusted mean active
Immediate Memory	1.567	0.222	0.057	87.21	93.21
Visuospatial Skill	0.002	0.967	0.0	99.27	99.5
Language	0.885	0.355	0.033	92.67	95.35
Attention	1.955	0.174	0.07	84.9	81.04
Delayed Memory	0.172	0.682	0.007	91.77	93.54
Total	1.366	0.253	0.05	87.21	90.21

Results of ANCOVA comparing post-treatment (V1) RBANS scores between the active and sham groups, controlling for baseline(V0) performance. Values represent adjusted means at the sample mean of baseline scores. F-statistics, p-values, and partial η² arereported for the main effect of group

### PANSS

#### *Baseline visit*

The difference between the active and sham groups at the baseline testing was not statistically significant in the total PANSS score (W = 66.5, p-value = 0.5477), the positive PANSS score (W = 86, *p* = 0.6796), nor the general PANSS score (t(20.22) = 1.2375, p-value = 0.2301). The difference was statistically significant in the negative PANSS score (W = 30, *p* = 0.009248).

#### *One-week endpoint visit*

No statistically significant difference in *total PANSS scores* was observed (t(22.05) = -1.35, p-value = 0.190, mean in sham group = -3.69, mean in active group = -1.58). No difference was observed between the groups in the change of the *negative PANSS scores* (t(19.959) = -0.013541, p-value = 0.9893, mean in sham group = -0.85, mean in active group = -0.83). A non-parametric comparison by Wilcoxon rank-sum test showed no significant difference between the groups in the *positive PANSS score* (W = 69.5, p-value = 0.6439, mean in sham group = -0.462, mean in active group = -0.250). In the *general PANSS score*, the Wilcoxon test indicated a statistically significant difference between the groups, favoring sham group (W = 38.5, p-value = 0.03095, mean in sham group = -2.385, mean in active group = -0.5).

### Three-week stimulation protocol

Eight patients opted to continue for 3 weeks of stimulation and cognitive training with preserved blinding of the randomization. Four patients continued in each group, active and sham. The results did not reveal a statistically significant difference in any of the RBANS or PANSS scores by the three-week endpoint. The results are available in the appendix.

### Tolerability and safety

The investigators asked participants about the tolerance of stimulation and any possible side effects after each application. The most frequently reported side effects were a tingling sensation or warmth under the stimulation electrodes and temporary redness of the skin at the stimulation site where the electrodes were positioned. The stimulation was overall well tolerated. No serious adverse effects occurred. At no point did the side effects result in discontinuation of the stimulation.

## Discussion

This randomized controlled study explored the combined intervention of tDCS and computer-based cognitive training aimed at ameliorating cognitive deficits associated with schizophrenia. A total of 30 subjects were included in the study; 29 enrolled patients completed the study, and the data were analyzed.

Although no statistically significant differences were detected, numerical trends across several RBANS domains (Total score, Memory, Visuoconstructional, and Language indices) were slightly higher in the active stimulation group. Surprisingly, the attention score was numerically higher in the sham group but also did not reach a statistically significant level. Additionally, no significant differences were observed in total PANSS scores or the positive and negative subscales between the groups; a significant group difference emerged in the general psychopathology subscale, favoring sham stimulation.

The observed trends in cognitive performance align with clinical expectations and the neuromodulation mechanisms of tDCS [29], especially when enhanced by concurrent cognitive training. Importantly, the domain-specific pattern of the results may reflect the specificity of engaged cortical regions in the selected electrode montage. While the left DLPFC, targeted in our study, is connected to working memory and higher-order analytical processing [61], its influence on attentional networks is more limited. Sustained attention has been linked to broader frontoparietal connectivity and right DLPFC [62, 63], areas not directly targeted by the applied tDCS montage. This distinction might clarify why positive trends were observed in memory-related domains, but not in attention.

In addition to the stimulation effects, the improvements in memory-related domains also correspond to the evidence linking cognitive training to increased activation of prefrontal and parietal regions [19–21]. Although cognitive tasks targeting attention were included in the protocol, as the stimulation did not directly target the regions associated with attention (particularly sustained and divided attention), the combination therapy may have preferentially enhanced memory-related processes. Future interventions may need to explore alternative or bilateral montages to engage attention-related circuits.

To date, only a limited number of studies have applied a combined intervention of tDCS and cognitive training delivered concurrently during each session in individuals with schizophrenia [43, 51–55]. Importantly, only a very recently published study incorporated the additional core elements required for cognitive remediation [55]. As highlighted in the critical review Lisoni et al. [64], the current studies predominantly include cognitive activation – repeated engagement in cognitive tasks – rather than cognitive remediation when pairing the interventions with tDCS. Across existing studies, the combination of tDCS with cognitive training brings heterogeneous results largely due to methodological variations [65]. Another key distinction emphasized by Lisoni et al. [64] concerns the intended interaction between interventions. tDCS may be combined with cognitive training to achieve synergistic effects when used together, or additive effects when used as a primer. Among the previously cited studies [43, 51–55], all applied tDCS concurrently with cognitive training to promote synergistic effects, and only one used an additional intervention immediately following tDCS, introducing a possible element of additive modulation [52]. These observations suggest that future research may benefit from integrating tDCS with fully evidence-based psychosocial interventions and from systematically exploring priming versus synergistic protocols, given the metaplastic-like effects of tDCS [66].

**Fig. 3:**
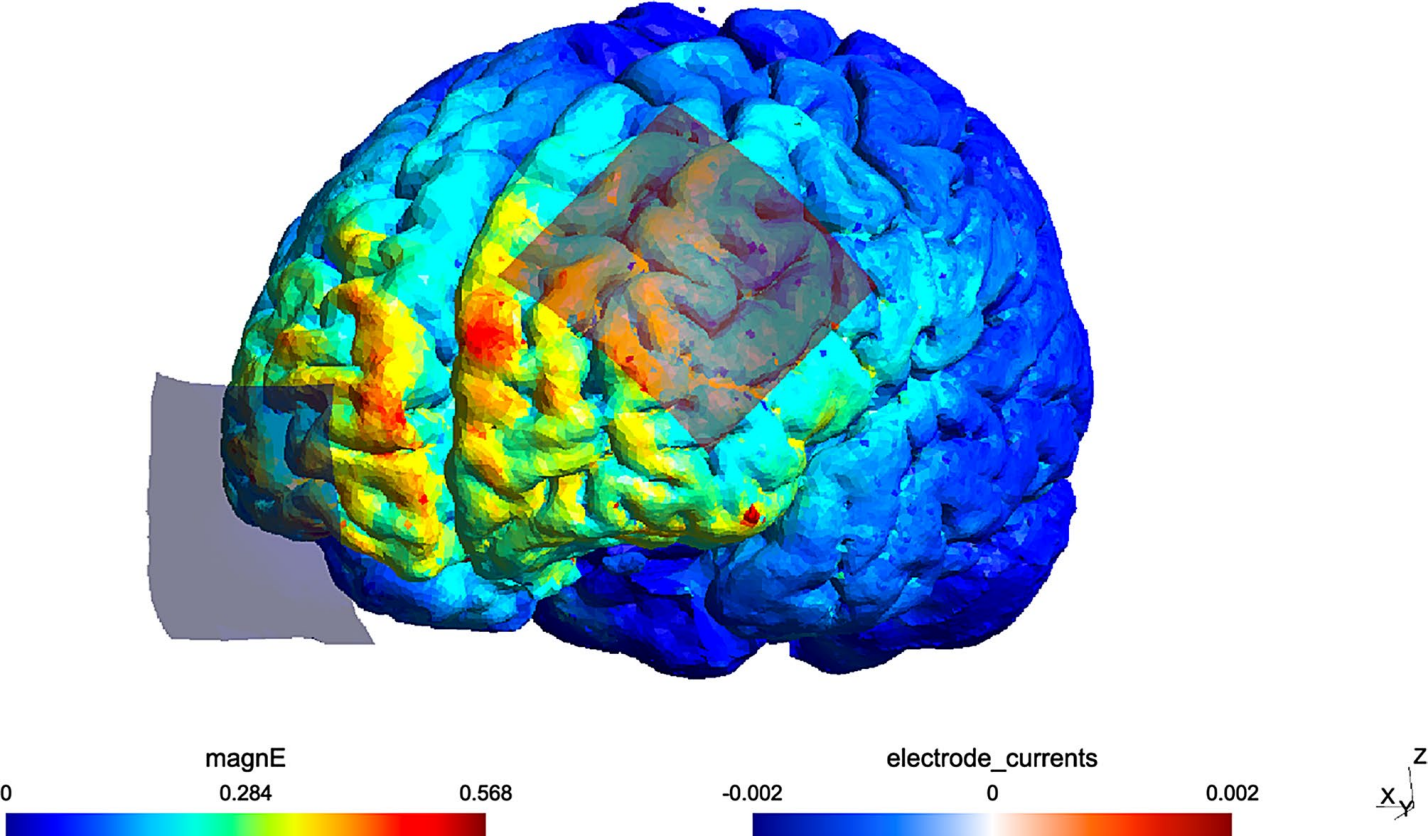
Electric field distribution during applied tDCS assembly. The computational modeling of the electric field was performed using SimNIBS 4.0 software; magnE is the electric field magnitude plotted in the gray matter surface, measured in V/m

Among the studies that applied a combined intervention of tDCS and cognitive training concurrently during each session in this population, four studies [52–54, 67] placed the anode over the F3 site to target DLPFC, and two of them used an electrode montage identical to the present study [52, 67]. Prior studies suggest cathode placement may be less essential for cognitive improvement when the anode is placed over the left DLPFC. However, although the DLPFC remains the most targeted region, alternative montages have also been explored, such as a more recent trial [54] with the stimulation of the posterior parietal cortex, an area also associated with working memory deficits in schizophrenia [68]. Our electrode placement was chosen based on studies targeting DLPFC with anode over F3 and cathode over Fp2. The electric field distribution was subsequently evaluated using the SimNIBS 4.0 simulator. The modeling, nonetheless, indicated that the stimulation predominantly affected the medial prefrontal cortex (mPFC), rather than the intended DLPFC (Fig. 3). This divergence may be due to differences in electrode characteristics. Specifically, variations in electrode shape (rectangular vs. square) and electrode orientation can significantly influence the targeted area, as observed in studies with similar montages [69]. In a previously mentioned study from this year where the SimNIBS simulator was used to determine the position of the electrodes to stimulate DLPFC, the anode was placed over C3 and the cathode over Fp1 [55]. The results of this study did, however, not reveal significant differences between sham and active tDCS when combined with cognitive remediation, likely due to a small sample size and expected improvements from the cognitive remediation alone. It is thus possible that a different electrode positioning in our protocol may have led to more favorable clinical outcomes in our study.

Regarding the stimulation duration, in the present study, tDCS was applied for 30 min per session. Current research suggests that the effect of anodal tDCS does not necessarily increase proportionally to the duration of the active stimulation and may even decrease or reverse with prolonged stimulation [70, 71]. Although most protocols select 20-minute stimulation when targeting cognition [39, 40, 51, 53, 72, 73], 30-minute stimulation was used in several previous studies with positive outcomes [36, 74].

Daily application was chosen to enhance the effects of stimulation, as supported by previous research [31, 75]. At the same time, stimulation frequency was selected once a day to ensure applicability in future clinical use and to facilitate the feasibility of the study protocol, as the study was conducted primarily in an outpatient setting. Repeated application protocols, however, offer an opportunity for induction of long-lasting effects and neuroplastic change [76].

### Strengths and limitations

We are aware of our study’s limitations. Firstly, the original study protocol was designed only for five following days. A longer stimulation seems to bring more prominent effects and might have been needed to show positive results of the stimulation, as neither active nor sham stimulation demonstrated statistically significant effects on cognition. Although some of the previously published studies showed a significant effect of tDCS on cognitive improvement after five applications [53, 54, 72], a recent meta-analysis in healthy adults noted that long-term interventions (≥ 10 sessions) with a stimulation intensity of 2 mA are the most effective [77]. For the three-week stimulation protocol, only eight patients opted to participate, which did not provide enough statistical power to detect any positive effects. Secondly, our study did not include a follow-up that might have separated the positive trends of the combined treatment from cognitive training alone to show whether the trends suggest a synergistic effect or an effect of only one of the methods. Many cognitive and neuroplastic effects associated with tDCS and cognitive training may accumulate or consolidate over time. Thirdly, there is a limitation concerning the nature of cognitive intervention. RehaCom is a software for computer-based cognitive rehabilitation, can be used as a component of a broader cognitive remediation program and was shown to be effective in enhancing cognition in schizophrenia in some studies [78, 79]. However, in our study, RehaCom was used to deliver structured cognitive training exercises. Our protocol did not include the additional core elements required for cognitive remediation – namely, therapist-guided strategy development and the incorporation of generalization procedures to support transfer of trained skills into everyday functioning. Fourthly, the sample size included in the study was small. This potentially reduced statistical power and increased the risk of type II errors. The sample size was determined based on a small number of previously published studies investigating similar interventions in this population. Recruitment was particularly challenging due to strict inclusion criteria. Lastly, although the study groups did not significantly differ in demographic data, heterogeneity was found in the illness duration as well as in the medication dose within the groups. Together with the variability in cognitive baseline, such heterogeneity can reduce the effect size and obscure findings. The differences between the cortical activity in early-course and chronic schizophrenia may be of consideration in connection to the anode placement. A previous study suggests a difference in the activity of the prefrontal cortex among early-course schizophrenic patients in contrast to attenuation of the activity that is commonly described in patients with chronic illness [80].

The strengths of our study include the following. This was a randomized controlled study with rigorous blinding. We used a standardized and repeatable cognitive assessment. As previously stated, only a limited number of studies applied the combination treatment of cognitive training and tDCS during each session. Thus, even with non-significant results, this study brings a contribution to a larger body of research and may be an indicator for a future direction of investigation. This is, to our knowledge, also the first randomized controlled study conducted in a Czech cohort to utilize this combined intervention approach in patients with schizophrenia. In addition, our study design shows good feasibility and tolerability, as seen in a low to no dropout rate.

To conclude, despite the limited sample size, the study employed rigorous methodology, including high-quality randomization, standardized interventions, and blinded outcome assessment, enhancing internal validity. These strengths, combined with the consistency of our results with prior findings, support the utility of our data as a foundation for future, larger-scale trials.

## Conclusion

To conclude, our findings do not provide evidence for the superiority of combined tDCS and cognitive training over cognitive training alone in patients with schizophrenia.

Numerical trends across several RBANS domains (Total Score, Memory, Visuoconstructional, and Language indices) were slightly higher in the active stimulation group, the attention score was numerically higher in the sham group, and no effects favoring active tDCS were found on the psychopathology scales. The numerical trends in memory-related domains suggest potential for benefit. However, these results should be interpreted with consideration of the short intervention duration, limited sample size, and absence of follow-up, which may have reduced the probability of detecting robust effects. Nevertheless, this study contributes to the growing body of research on neuromodulation in schizophrenia, highlighting both the promise and the practical challenges of implementing combined therapeutic approaches in real-world clinical populations. Larger and longer trials would be beneficial to clarify the clinical utility of this intervention strategy.

## Supplementary Information

Below is the link to the electronic supplementary material.

Supplementary Material 1

## Data Availability

The datasets used and/or analyzed during the current study are available from the corresponding author on reasonable request.

## Abbreviations

BDNF: Brain–Derived Neurotropic Factor
DLPFC: Dorsolateral Prefrontal Cortex
DSM: Diagnostic and Statistical Manual of Mental Disorders
GABA: Gamma–Aminobutyric Acid
ICD: 10–International Statistical Classification of Diseases and Related Health Problems 10th Revision
mPFC: medial Prefrontal Cortex
NIMH: National Institute of Mental Health
PANSS: Positive and Negative Syndrome Scale
RBANS: Repeatable Battery for the Assessment of Neuropsychological Status
tDCS: transcranial Direct Current Stimulation

## References

[1] McCutcheon RA, Keefe RSE, McGuire PK. Cognitive impairment in schizophrenia: aetiology, pathophysiology, and treatment. Mol Psychiatry. 2023;28(5):1902–18. 36690793 10.1038/s41380-023-01949-9 PMC10575791

[2] Bozikas VP, Andreou C. Longitudinal studies of cognition in first episode psychosis: a systematic review of the literature. Aust N Z J Psychiatry. 2011;45(2):93–108. 21320033 10.3109/00048674.2010.541418

[3] Fioravanti M, Bianchi V, Cinti ME. Cognitive deficits in schizophrenia: an updated metanalysis of the scientific evidence. BMC Psychiatry. 2012;12:64. 22715980 10.1186/1471-244X-12-64 PMC3528440

[4] Keefe RSE, Harvey PD. Cognitive Impairment in Schizophrenia. In: Geyer MA, Gross G, editors. Novel Antischizophrenia Treatments [Internet]. Berlin, Heidelberg: Springer Berlin Heidelberg; 2012 [cited 2023 Oct 30]. pp. 11–37. (Handbook of Experimental Pharmacology; vol. 213). Available from: http://link.springer.com/10.1007/978-3-642-25758-2_2 10.1007/978-3-642-25758-2_2 23027411

[5] Deste G, Vita A, Nibbio G, Penn DL, Pinkham AE, Harvey PD. Autistic Symptoms and Social Cognition Predict Real-World Outcomes in Patients With Schizophrenia. Front Psychiatry [Internet]. 2020 Jun 5 [cited 2025 Apr 3];11. Available from: https://www.frontiersin.org/journals/psychiatry/articles/10.3389/fpsyt.2020.00524/full 10.3389/fpsyt.2020.00524 PMC7294984 32581892

[6] Baldez DP, Biazus TB, Rabelo-da-Ponte FD, Nogaro GP, Martins DS, Kunz M, et al. The effect of antipsychotics on the cognitive performance of individuals with psychotic disorders: network meta-analyses of randomized controlled trials. Neurosci Biobehavioral Reviews. 2021;126:265–75. 10.1016/j.neubiorev.2021.03.028 33812977

[7] Andreasen NC, O’Leary DS, Flaum M, Nopoulos P, Watkins GL, Boles Ponto LL, et al. Hypofrontality in schizophrenia: distributed dysfunctional circuits in neuroleptic-naïve patients. Lancet. 1997;349(9067):1730–4. 9193383 10.1016/s0140-6736(96)08258-x

[8] Hill K, Mann L, Laws KR, Stephenson CME, Nimmo-Smith I, McKenna PJ. Hypofrontality in schizophrenia: a meta-analysis of functional imaging studies. Acta Psychiatrica Scandinavica. 2004;110(4):243–56. 15352925 10.1111/j.1600-0447.2004.00376.x

[9] Schmitt A, Hasan A, Gruber O, Falkai P. Schizophrenia as a disorder of disconnectivity. Eur Arch Psychiatry Clin Neurosci. 2011;261(Suppl 2):S150–154. 21866371 10.1007/s00406-011-0242-2 PMC3207137

[10] Glahn DC, Ragland JD, Abramoff A, Barrett J, Laird AR, Bearden CE, et al. Beyond hypofrontality: a quantitative meta-analysis of functional neuroimaging studies of working memory in schizophrenia. Hum Brain Mapp. 2005;25(1):60–9. 15846819 10.1002/hbm.20138 PMC6871703

[11] Adhikari BM, Hong LE, Sampath H, Chiappelli J, Jahanshad N, Thompson PM, et al. Functional network connectivity impairments and core cognitive deficits in schizophrenia. Hum Brain Mapp. 2019;40(16):4593–605. 31313441 10.1002/hbm.24723 PMC6865503

[12] Li YT, Zhang C, Han JC, Shang YX, Chen ZH, Cui GB, et al. Neuroimaging features of cognitive impairments in schizophrenia and major depressive disorder. Therapeutic Adv Psychopharmacol. 2024;14:20451253241243290. 10.1177/20451253241243290 PMC11070126 38708374

[13] Simeone JC, Ward AJ, Rotella P, Collins J, Windisch R. An evaluation of variation in published estimates of schizophrenia prevalence from 19902013: a systematic literature review. BMC Psychiatry. 2015;15(1):193. 26263900 10.1186/s12888-015-0578-7 PMC4533792

[14] Gillespie KM, Dymond AH, Li X, Schweitzer D, Branjerdporn G, Khan S, et al. A systematic review and narrative synthesis of cognitive training in the treatment of mental illness and substance use disorder. J Clin Med. 2024;13(15):4348. 39124616 10.3390/jcm13154348 PMC11312778

[15] Bowie CR, Bell MD, Fiszdon JM, Johannesen JK, Lindenmayer JP, McGurk SR, et al. Cognitive remediation for schizophrenia: an expert working group white paper on core techniques. Schizophr Res. 2020;215:49–53. 31699627 10.1016/j.schres.2019.10.047

[16] McGurk SR, Twamley EW, Sitzer DI, McHugo GJ, Mueser KT. A Meta-Analysis of cognitive remediation in schizophrenia. Am J Psychiatry. 2007;164(12):1791–802. 18056233 10.1176/appi.ajp.2007.07060906 PMC3634703

[17] Wykes T, Huddy V, Cellard C, McGurk SR, Czobor P. A Meta-Analysis of cognitive remediation for schizophrenia: methodology and effect sizes. AJP. 2011;168(5):472–85. 10.1176/appi.ajp.2010.10060855 21406461

[18] Zoupa E, Bogiatzidou O, Siokas V, Liampas I, Tzeferakos G, Mavreas V, et al. Cognitive rehabilitation in Schizophrenia-Associated cognitive impairment: A review. Neurol Int. 2022;15(1):12–23. 36648966 10.3390/neurolint15010002 PMC9844333

[19] Haut KM, Lim KO, MacDonald A. Prefrontal cortical changes following cognitive training in patients with chronic schizophrenia: effects of Practice, Generalization, and specificity. Neuropsychopharmacol. 2010;35(9):1850–9. 10.1038/npp.2010.52 PMC3055638 20428109

[20] Wykes T, Brammer M, Mellers J, Bray P, Reeder C, Williams C, et al. Effects on the brain of a psychological treatment: cognitive remediation therapy: functional magnetic resonance imaging in schizophrenia. Br J Psychiatry. 2002;181(2):144–52. 12151286 10.1017/s0007125000161872

[21] Rowland LM, Griego JA, Spieker EA, Cortes CR, Holcomb HH. Neural changes associated with relational learning in schizophrenia. Schizophr Bull. 2010;36(3):496–503. 20418447 10.1093/schbul/sbq037 PMC2879675

[22] Eack SM, Newhill CE, Keshavan MS. Cognitive enhancement therapy improves Resting-State functional connectivity in early course schizophrenia. J Soc Social Work Res. 2016;7(2):211–30. 27713804 10.1086/686538 PMC5047289

[23] Donohoe G, Dillon R, Hargreaves A, Mothersill O, Castorina M, Furey E, et al. Effectiveness of a low support, remotely accessible, cognitive remediation training programme for chronic psychosis: cognitive, functional and cortical outcomes from a single blind randomised controlled trial. Psychol Med. 2018;48(5):751–64. 28933314 10.1017/S0033291717001982

[24] Subramaniam K, Gill J, Fisher M, Mukherjee P, Nagarajan S, Vinogradov S. White matter microstructure predicts cognitive training-induced improvements in attention and executive functioning in schizophrenia. Schizophr Res. 2018;193:276–83. 28689758 10.1016/j.schres.2017.06.062 PMC5999406

[25] Penadés R, Pujol N, Catalán R, Massana G, Rametti G, García-Rizo C, et al. Brain effects of cognitive remediation therapy in schizophrenia: A structural and functional neuroimaging study. Biol Psychiatry. 2013;73(10):1015–23. 23452665 10.1016/j.biopsych.2013.01.017

[26] Morimoto T, Matsuda Y, Matsuoka K, Yasuno F, Ikebuchi E, Kameda H, et al. Computer-assisted cognitive remediation therapy increases hippocampal volume in patients with schizophrenia: a randomized controlled trial. BMC Psychiatry. 2018;18(1):83. 29587688 10.1186/s12888-018-1667-1 PMC5870916

[27] Eack SM, Hogarty GE, Cho RY, Prasad KMR, Greenwald DP, Hogarty SS, et al. Cognitive enhancement therapy protects against Gray matter loss in early schizophrenia: results from a Two-Year randomized controlled trial. Arch Gen Psychiatry. 2010;67(7):674–82. 20439824 10.1001/archgenpsychiatry.2010.63 PMC3741671

[28] Mothersill D, Donohoe G. Neural effects of cognitive training in schizophrenia: A systematic review and activation likelihood Estimation Meta-analysis. Biol Psychiatry: Cogn Neurosci Neuroimaging. 2019;4(8):688–96. 31072761 10.1016/j.bpsc.2019.03.005

[29] Lefaucheur JP, Antal A, Ayache SS, Benninger DH, Brunelin J, Cogiamanian F, et al. Evidence-based guidelines on the therapeutic use of transcranial direct current stimulation (tDCS). Clin Neurophysiol. 2017;128(1):56–92. 27866120 10.1016/j.clinph.2016.10.087

[30] Fregni F, El-Hagrassy MM, Pacheco-Barrios K, Carvalho S, Leite J, Simis M, et al. Evidence-Based guidelines and secondary Meta-Analysis for the use of transcranial direct current stimulation in neurological and psychiatric disorders. Int J Neuropsychopharmacol. 2021;24(4):256–313. 32710772 10.1093/ijnp/pyaa051 PMC8059493

[31] Hyde J, Carr H, Kelley N, Seneviratne R, Reed C, Parlatini V, et al. Efficacy of neurostimulation across mental disorders: systematic review and meta-analysis of 208 randomized controlled trials. Mol Psychiatry. 2022;27(6):2709–19. 35365806 10.1038/s41380-022-01524-8 PMC8973679

[32] Brunoni AR, Nitsche MA, Bolognini N, Bikson M, Wagner T, Merabet L, et al. Clinical research with transcranial direct current stimulation (tDCS): challenges and future directions. Brain Stimul. 2012;5(3):175–95. 22037126 10.1016/j.brs.2011.03.002 PMC3270156

[33] Nitsche MA, Cohen LG, Wassermann EM, Priori A, Lang N, Antal A, et al. Transcranial direct current stimulation: state of the Art 2008. Brain Stimul. 2008;1(3):206–23. 20633386 10.1016/j.brs.2008.06.004

[34] Fritsch B, Reis J, Martinowich K, Schambra HM, Ji Y, Cohen LG, et al. Direct current stimulation promotes BDNF-dependent synaptic plasticity: potential implications for motor learning. Neuron. 2010;66(2):198–204. 20434997 10.1016/j.neuron.2010.03.035 PMC2864780

[35] To WT, De Ridder D, Hart J Jr., Vanneste S. Changing Brain Networks Through Non-invasive Neuromodulation. Front Hum Neurosci [Internet]. 2018 Apr 13 [cited 2025 Apr 5];12. Available from: https://www.frontiersin.org/journals/human-neuroscience/articles/10.3389/fnhum.2018.00128/full 10.3389/fnhum.2018.00128 PMC5908883 29706876

[36] Orlov ND, Tracy DK, Joyce D, Patel S, Rodzinka-Pasko J, Dolan H, et al. Stimulating cognition in schizophrenia: A controlled pilot study of the effects of prefrontal transcranial direct current stimulation upon memory and learning. Brain Stimul. 2017;10(3):560–6. 28057452 10.1016/j.brs.2016.12.013

[37] Lisoni J, Baldacci G, Nibbio G, Zucchetti A, Butti Lemmi Gigli E, Savorelli A, et al. Effects of bilateral, bipolar-nonbalanced, frontal transcranial direct current stimulation (tDCS) on negative symptoms and neurocognition in a sample of patients living with schizophrenia: results of a randomized double-blind sham-controlled trial. J Psychiatr Res. 2022;155:430–42. 36182772 10.1016/j.jpsychires.2022.09.011

[38] Nienow TM, MacDonald AW, Lim KO. TDCS produces incremental gain when combined with working memory training in patients with schizophrenia: A proof of concept pilot study. Schizophr Res. 2016;172(1):218–9. 26852404 10.1016/j.schres.2016.01.053

[39] Smith RC, Md WL, Wang Y, Jiang J, Wang J, Szabo V, et al. Effects of transcranial direct current stimulation on cognition and symptoms in Chinese patients with schizophrenia. Psychiatry Res. 2020;284:112617. 31806403 10.1016/j.psychres.2019.112617

[40] García-Fernández L, Romero-Ferreiro V, Padilla S, Wynn R, Pérez-Gálvez B, Álvarez-Mon MÁ, et al. Transcranial direct current stimulation (tDCS) enhances cognitive function in schizophrenia: A randomized double-blind sham-controlled trial. Psychiatry Res. 2025;344:116308. 39647260 10.1016/j.psychres.2024.116308

[41] Shiozawa P, Gomes JS, Ducos DV, Akiba HT, Dias ÁM, Trevizol AP, et al. Effect of transcranial direct current stimulation (tDCS) over the prefrontal cortex combined with cognitive training for treating schizophrenia: a sham-controlled randomized clinical trial. Trends Psychiatry Psychother. 2016;38(3):175–7. 27737311 10.1590/2237-6089-2015-0043

[42] Gomes JS, Trevizol AP, Ducos DV, Gadelha A, Ortiz BB, Fonseca AO, et al. Effects of transcranial direct current stimulation on working memory and negative symptoms in schizophrenia: a phase II randomized sham-controlled trial. Schizophrenia Research: Cognition. 2018;12:20–8. 29552509 10.1016/j.scog.2018.02.003 PMC5852322

[43] Schilling TM, Andelfinger V, Bossert M, König M, Wolfenson D, Lang S, et al. No clinically relevant effects of 12 sessions of 2 mA of anodal transcranial direct current stimulation over the left DLPFC in combination with concurrent cognitive training compared to cognitive training only on executive functions in patients with schizophrenia - A randomized controlled trial. Schizophr Res. 2022;248:287–9. 36122444 10.1016/j.schres.2022.09.002

[44] Zhou Y, Xia X, Zhao X, Yang R, Wu Y, Liu J, et al. Efficacy and safety of transcranial direct current stimulation (tDCS) on cognitive function in chronic schizophrenia with tardive dyskinesia (TD): a randomized, double-blind, sham-controlled, clinical trial. BMC Psychiatry. 2023;23(1):623. 37620825 10.1186/s12888-023-05112-0 PMC10464035

[45] Li X, Dai J, Liu Q, Zhao Z, Zhang X. Efficacy and safety of non-invasive brain stimulation on cognitive function for cognitive impairment associated with schizophrenia: A systematic review and meta-analysis. J Psychiatr Res. 2024;170:174–86. 38150769 10.1016/j.jpsychires.2023.12.003

[46] Safwi SR, Rizvi A, Usmani MA, Husain K, Brar K, Yadava D. Transcranial direct current stimulation and its effect on cognitive symptoms of schizophrenia: an updated review. Schizophrenia Research: Cognition. 2025;39:100335. 39512786 10.1016/j.scog.2024.100335 PMC11541428

[47] Segrave RA, Arnold S, Hoy K, Fitzgerald PB. Concurrent cognitive control training augments the antidepressant efficacy of tDCS: a pilot study. Brain Stimul. 2014;7(2):325–31. 24486425 10.1016/j.brs.2013.12.008

[48] Pallanti S, Grassi E, Knotkova H, Galli G. Transcranial direct current stimulation in combination with cognitive training in individuals with mild cognitive impairment: a controlled 3-parallel-arm study. CNS Spectr. 2023;28(4):489–94. 36093863 10.1017/S1092852922000979

[49] Kazinka R, Roediger D, Xuan L, Yu L, Mueller BA, Camchong J, et al. tDCS-enhanced cognitive training improves attention and alters connectivity in control and somatomotor networks: A triple blind study. NeuroImage. 2024;298:120792. 39147294 10.1016/j.neuroimage.2024.120792 PMC11425656

[50] Burton CZ, Garnett EO, Capellari E, Chang SE, Tso IF, Hampstead BM, et al. Combined cognitive training and transcranial direct current stimulation in neuropsychiatric disorders: A systematic review and Meta-analysis. Biol Psychiatry Cogn Neurosci Neuroimaging. 2023;8(2):151–61. 36653210 10.1016/j.bpsc.2022.09.014 PMC10823589

[51] Weickert TW, Salimuddin H, Lenroot RK, Bruggemann J, Loo C, Vercammen A, et al. Preliminary findings of four-week, task-based anodal prefrontal cortex transcranial direct current stimulation transferring to other cognitive improvements in schizophrenia. Psychiatry Res. 2019;280:112487. 31376788 10.1016/j.psychres.2019.112487

[52] Fathi Azar E, Hosseinzadeh S, Nosrat Abadi M, Sayad Nasiri M, Haghgoo HA. Impact of psychosocial occupational therapy combined with anodal transcranial direct current stimulation to the left dorsolateral prefrontal cortex on the cognitive performance of patients with schizophrenia: A randomized controlled trial. Hong Kong J Occup Therapy. 2021;34(2):121–31. 10.1177/15691861211065155 PMC8721578 34987350

[53] Lo KYH, Hopman HJ, Chan SC, Chau WHS, Cheng PWC, Cheung KY, et al. Concurrent anodal transcranial direct current stimulation (tDCS) with cognitive training to improve cognition in schizophrenia. Schizophr Res. 2022;241:184–6. 35131597 10.1016/j.schres.2022.01.026

[54] Hou W, Zhou F, Wang Q, Li H, Qin X, Ding Y, et al. Effect of transcranial direct current stimulation with concurrent cognitive performance targeting posterior parietal cortex vs prefrontal cortex on working memory in schizophrenia: a randomized clinical trial. Transl Psychiatry. 2024;14(1):279. 38977683 10.1038/s41398-024-02994-w PMC11231223

[55] Poppe A, Bais L, van Duin D, Ćurčić-Blake B, Pijnenborg GHM, van der Meer L. Feasibility and acceptability of combining cognitive remediation and tDCS in long-term psychiatric clinical care. Schizophr Res Cogn. 2025;42:100358. 40551882 10.1016/j.scog.2025.100358 PMC12182772

[56] Leucht S, Kane JM, Kissling W, Hamann J, Etschel E, Engel RR. What does the PANSS mean? Schizophr Res. 2005;79(2):231–8. 15982856 10.1016/j.schres.2005.04.008

[57] Kašpárek T, Ustohal L. Akutní léčba schizofrenie. Doporučené postupy psychiatrické péče 2023 [Internet]. 2023 [cited 2025 Dec 26]. Available from: https://postupy-pece.psychiatrie.cz/images/pdf/2023_DP_Akutni-lecba-schizofrenie_autorUstohal_oponentMohr.pdf.

[58] Randolph C, Tierney MC, Mohr E, Chase TN. The repeatable battery for the assessment of neuropsychological status (RBANS): preliminary clinical validity. J Clin Exp Neuropsychol. 1998;20(3):310–9. 9845158 10.1076/jcen.20.3.310.823

[59] Randolph C. Repeatable Battery for the Assessment of Neuropsychological Status [Internet]. 2012 [cited 2025 Sep 4]. Available from: 10.1037/t15149-000

[60] Kay SR, Fiszbein A, Opler LA. The positive and negative syndrome scale (PANSS) for schizophrenia. Schizophr Bull. 1987;13(2):261–76. 3616518 10.1093/schbul/13.2.261

[61] Curtis CE, D’Esposito M. Persistent activity in the prefrontal cortex during working memory. Trends Cogn Sci. 2003;7(9):415–23. 12963473 10.1016/s1364-6613(03)00197-9

[62] Petersen SE, Posner MI. The attention system of the human brain: 20 years after. Annu Rev Neurosci. 2012;35(1):73–89. 22524787 10.1146/annurev-neuro-062111-150525 PMC3413263

[63] Sanchez A, Vanderhasselt MA, Baeken C, De Raedt R. Effects of tDCS over the right DLPFC on attentional disengagement from positive and negative faces: an eye-tracking study. Cogn Affect Behav Neurosci. 2016;16(6):1027–38. 27495805 10.3758/s13415-016-0450-3

[64] Lisoni J, Nibbio G, Baglioni A, Dini S, Manera B, Maccari A, et al. Is it possible to combine Non-Invasive brain stimulation and Evidence-Based psychosocial interventions in schizophrenia? A critical review. Brain Sci. 2024;14(11):1067. 39595830 10.3390/brainsci14111067 PMC11591595

[65] Poppe A, Ritter FDE, Bais L, Pustejovsky JE, van Tol MJ, Ćurčić-Blake B, et al. The efficacy of combining cognitive training and noninvasive brain stimulation: A transdiagnostic systematic review and meta-analysis. Psychol Bull. 2024;150(2):192–213. 37956054 10.1037/bul0000406

[66] Hurley R, Machado L. Using tDCS priming to improve brain function: can metaplasticity provide the key to boosting outcomes? Neurosci Biobehav Rev. 2017;83:155–9. 29020606 10.1016/j.neubiorev.2017.09.029

[67] Schilling TM, Bossert M, König M, Wirtz G, Weisbrod M, Aschenbrenner S. Acute effects of a single dose of 2 mA of anodal transcranial direct current stimulation over the left dorsolateral prefrontal cortex on executive functions in patients with schizophrenia-A randomized controlled trial. PLoS ONE. 2021;16(7):e0254695. 34270620 10.1371/journal.pone.0254695 PMC8284793

[68] Hahn B, Robinson BM, Leonard CJ, Luck SJ, Gold JM. Posterior parietal cortex dysfunction is central to working memory storage and broad cognitive deficits in schizophrenia. J Neurosci. 2018;38(39):8378–87. 30104335 10.1523/JNEUROSCI.0913-18.2018 PMC6158696

[69] Oliver-Mas S, Delgado-Alonso C, Delgado-Álvarez A, Díez-Cirarda M, Cuevas C, Fernández-Romero L, et al. Transcranial direct current stimulation for post-COVID fatigue: a randomized, double-blind, controlled pilot study. Brain Commun. 2023;5(2):fcad117. 37091591 10.1093/braincomms/fcad117 PMC10116605

[70] Vignaud P, Mondino M, Poulet E, Palm U, Brunelin J. Duration but not intensity influences transcranial direct current stimulation (tDCS) after-effects on cortical excitability. Neurophysiol Clin. 2018;48(2):89–92. 29482881 10.1016/j.neucli.2018.02.001

[71] Hassanzahraee M, Nitsche MA, Zoghi M, Jaberzadeh S. Determination of anodal tDCS intensity threshold for reversal of corticospinal excitability: an investigation for induction of counter-regulatory mechanisms. Sci Rep. 2020;10(1):16108. 32999375 10.1038/s41598-020-72909-4 PMC7527486

[72] Smith RC, Boules S, Mattiuz S, Youssef M, Tobe RH, Sershen H, et al. Effects of transcranial direct current stimulation (tDCS) on cognition, symptoms, and smoking in schizophrenia: A randomized controlled study. Schizophr Res. 2015;168(1):260–6. 26190299 10.1016/j.schres.2015.06.011

[73] Meiron O, David J, Yaniv A. Left prefrontal transcranial direct-current stimulation reduces symptom-severity and acutely enhances working memory in schizophrenia. Neurosci Lett. 2021;755:135912. 33894334 10.1016/j.neulet.2021.135912

[74] Jeon DW, Jung DU, Kim SJ, Shim JC, Moon JJ, Seo YS, et al. Adjunct transcranial direct current stimulation improves cognitive function in patients with schizophrenia: A double-blind 12-week study. Schizophr Res. 2018;197:378–85. 30955702 10.1016/j.schres.2017.12.009

[75] Alonzo A, Brassil J, Taylor JL, Martin D, Loo CK. Daily transcranial direct current stimulation (tDCS) leads to greater increases in cortical excitability than second daily transcranial direct current stimulation. Brain Stimul. 2012;5(3):208–13. 22037139 10.1016/j.brs.2011.04.006

[76] Goldsworthy MR, Pitcher JB, Ridding MC. Spaced noninvasive brain stimulation: prospects for inducing Long-Lasting human cortical plasticity. Neurorehabil Neural Repair. 2015;29(8):714–21. 25505220 10.1177/1545968314562649

[77] Lv Y, Wu S, Nitsche MA, Yue T, Zschorlich VR, Qi F. A meta-analysis of the effects of transcranial direct current stimulation combined with cognitive training on working memory in healthy older adults. Front Aging Neurosci. 2024;16:1454755. 39376507 10.3389/fnagi.2024.1454755 PMC11456488

[78] Yamanushi A, Shimada T, Koizumi A, Kobayashi M. Effect of Computer-Assisted cognitive remediation therapy on cognition among patients with schizophrenia: A pilot randomized controlled trial. Biomedicines. 2024;12(7):1498. 39062072 10.3390/biomedicines12071498 PMC11274551

[79] Mak M, Samochowiec J, Tybura P, Bieńkowski P, Karakiewicz B, Zaremba Pechmann L, et al. The efficacy of cognitive rehabilitation with rehacom programme in schizophrenia patients. The role of selected genetic polymorphisms in successful cognitive rehabilitation. Ann Agric Environ Med. 2013;20(1):77–81. 23540216

[80] Anticevic A, Hu X, Xiao Y, Hu J, Li F, Bi F, et al. Early-Course unmedicated schizophrenia patients exhibit elevated prefrontal connectivity associated with longitudinal change. J Neurosci. 2015;35(1):267–86. 25568120 10.1523/JNEUROSCI.2310-14.2015 PMC4287147

