# Supplementary material for: Transcranial direct current stimulation and cognitive training in the treatment of cognitive deficit in schizophrenia: a randomized controlled trial
Source: BMC Psychiatry. 2026 Jan 6;26:102. doi: 10.1186/s12888-025-07749-5 (PMC12857147; doi:10.1186/s12888-025-07749-5)
Supplement: Supplementary file 1 — Supplementary Material 1 [file 12888_2025_7749_MOESM1_ESM.pdf]

Appendix. Results 3 week end point

variable		time	.y.	group 1	group 2	n1	n2	statistic	p	p.adj	p.adj.signif
PANSS	General	baseline	value	sham	active	4	4	11.5	0.381	0.6885	ns
		1 week	value	sham	active	4	4	10.5	0.552	0.6885	ns
		3 week	value	sham	active	4	4	10.5	0.561	0.6885	ns
	Negative	baseline	value	sham	active	4	4	5.5	0.561	0.6885	ns
		1 week	value	sham	active	4	4	3.5	0.231	0.6885	ns
		3 week	value	sham	active	4	4	3.5	0.231	0.6885	ns
	Positive	baseline	value	sham	active	4	4	8.5	1	1	ns
		1 week	value	sham	active	4	4	8.5	1	1	ns
		3 week	value	sham	active	4	4	9.5	0.766	0.83376	ns
RBANS	Immediate Memory	baseline	value	sham	active	4	4	11.5	0.384	0.6885	ns
		1 week	value	sham	active	4	4	10.5	0.559	0.6885	ns
		3 week	value	sham	active	4	4	10	0.686	0.805304347826087	ns
	Visuospacial Skill	baseline	value	sham	active	4	4	9.5	0.772	0.83376	ns
		1 week	value	sham	active	4	4	12.5	0.245	0.6885	ns
		3 week	value	sham	active	4	4	11	0.462	0.6885	ns
	Delayed Memory	baseline	value	sham	active	4	4	11.5	0.384	0.6885	ns
		1 week	value	sham	active	4	4	14	0.11	0.6885	ns
		3 week	value	sham	active	4	4	11	0.468	0.6885	ns
	Attention	baseline	value	sham	active	4	4	10.5	0.561	0.6885	ns
		1 week	value	sham	active	4	4	11.5	0.384	0.6885	ns
		3 week	value	sham	active	4	4	12	0.343	0.6885	ns
	Language	baseline	value	sham	active	4	4	14	0.104	0.6885	ns
		1 week	value	sham	active	4	4	3	0.189	0.6885	ns
		3 week	value	sham	active	4	4	12	0.309	0.6885	ns
	Total	baseline	value	sham	active	4	4	14	0.114	0.6885	ns
		1 week	value	sham	active	4	4	13	0.2	0.6885	ns
		3 week	value	sham	active	4	4	13	0.2	0.6885	ns

PANSS – Positive and Negative Syndrome Scale; RBANS – Repeatable Battery for the Assessment of Neurological Status
